# Primary Small Cell Carcinoma of the Kidney: Disease Characteristics and Treatment Outcomes

**DOI:** 10.3390/medicines8010006

**Published:** 2021-01-18

**Authors:** Thomas F. Monaghan, Kyle P. Michelson, Nicholas R. Suss, Christina W. Agudelo, Syed N. Rahman, Dennis J. Robins, Viktor X. Flores, Brian K. McNeil, Jeffrey P. Weiss, Andrew G. Winer

**Affiliations:** 1 Department of Urology, SUNY Downstate Health Sciences University, 450 Clarkson Ave, P.O. Box 79, Brooklyn, NY 11203, USA; kyle.p.michelson@gmail.com (K.P.M.); nicholas.suss@downstate.edu (N.R.S.); cwagudelo@gmail.com (C.W.A.); syed.rahman@downstate.edu (S.N.R.); dennis.robins@downstate.edu (D.J.R.); viktor.flores@downstate.edu (V.X.F.); brian.mcneil@downstate.edu (B.K.M.); jeffrey.weiss@downstate.edu (J.P.W.); andrew.winer@downstate.edu (A.G.W.); 2 Division of Urology, Kings County Hospital Center, Brooklyn, NY 11203, USA; 3 Department of Urology, University of South Florida, Tampa, FL 33606, USA

**Keywords:** extrapulmonary, National Cancer Database, oncology, renal, small cell carcinoma (SCC), urology

## Abstract

**Background:** Primary small cell carcinoma of the kidney (PSCCK) is exceedingly rare and data on disease characteristics and outcomes are sparse. This study examines a nationally-representative cancer registry to better characterize PSCCK. **Methods:** We queried the National Cancer Database to identify patients with histology-confirmed PSCCK from 2004 to 2015. Adjusted Cox proportional hazards regression and Kaplan–Meier analyses were employed to assess predictors of mortality and estimate median survival time, respectively. **Results:** A total of 110 patients were included (47:53% female:male, 77% ≥60 years of age, 86% Caucasian). Significant predictors of mortality included female sex, age 60–69 years, treatment at an Integrated Network Cancer Program, stage cM1, and lack of surgical and chemoradiotherapy treatment. Independent protective factors were high socioeconomic status and treatment at an Academic Research Program. The estimated median overall survival time was 9.31 (95% CI 7.28–10.98) months for all patients. No differences in estimated survival time were observed across individual treatment modalities among those patients who underwent treatment (*p* = 0.214). **Conclusions:** PSCCK is an aggressive malignancy with a median survival time of less than one year. Future studies that correlate clinical tumor staging with specific treatment modalities are needed to optimize and individualize management.

## 1. Introduction

Extrapulmonary small cell carcinoma (SCC) is a rare entity, comprising 2.5% of all small cell malignancies [1], and primary SCC of the kidney (PSCCK) constitutes but a fraction of all extrapulmonary SCCs. PSCCK has been hypothesized to arise from enterochromaffin cells embedded within the urinary tract [2], metaplasia of high-grade malignancy [3,4,5], or aberrant differentiation of local pluripotent stem cells [6,7,8,9]. Although the etiology of PSCCK remains poorly understood, recent advances in immunohistochemical techniques carry the potential to improve the diagnostic sensitivity for PSCCK [10], and practice trends favoring active surveillance and biopsies of renal masses also stand to increase the observed incidence of PSCCK. As such, it will be increasingly important to familiarize clinicians with this malignancy, particularly in view of the fact that the presenting signs of PSCCK greatly mimic those of renal cell carcinoma [11,12,13,14]. To date, however, literature on PSCCK is largely limited to single-institution analyses, small case series, and expert opinion. Accordingly, this study aims to elucidate disease characteristics, predictors of mortality, and treatment outcomes using nationally-representative data from the United States.

## 2. Materials and Methods

### 2.1. Data Source

The National Cancer Database (NCDB) is a facility-based, nationwide cancer registry that captures more than 70% of all annual invasive cancer diagnoses in the United States. Patient-level demographics, facility characteristics, cancer-specific information, and treatment modalities are available from upwards of 1500 institutions participating in the American College of Surgeons Commission on Cancer approvals program [15]. Institutional Review Board approval was not required because the NCDB is widely accessible and deidentifies all patient, provider, and hospital information in accordance with Health Insurance Portability and Accountability Act privacy standards. The American College of Surgeons Commission on Cancer is not responsible for the study design, methodological plan, or interpretation of results presented herein and has not verified the conclusions drawn by investigators.

### 2.2. Patient Selection

**Figure 1:**
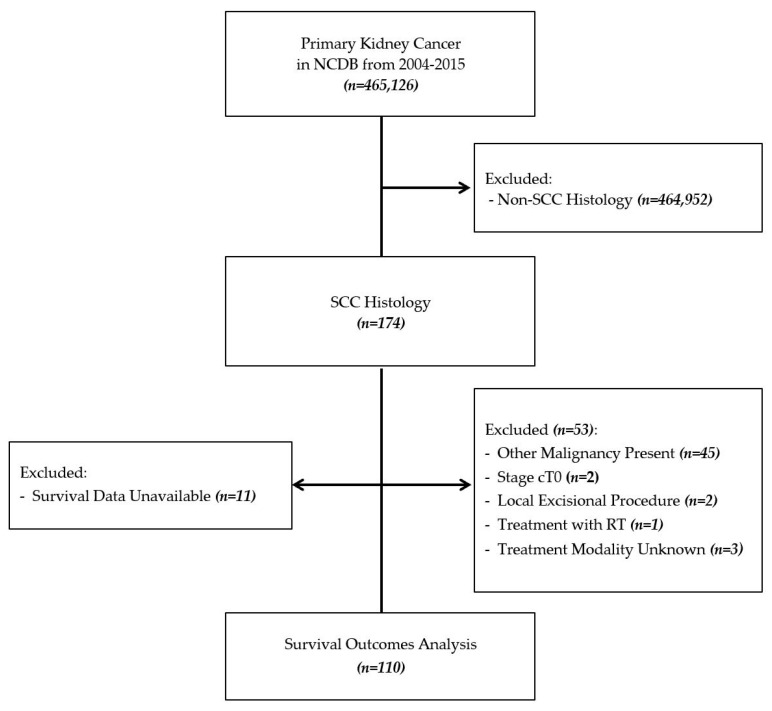
Selection map.

The NCDB was queried to identify and characterize patients with histology-confirmed PSCCK between 2004 and 2015. Patients with a concurrent diagnosis of any other primary malignancy, no identifiable primary tumor, or who were designated as clinical tumor (cT) stage cT0 were excluded from the analysis (Figure 1). Patients who underwent a local surgical excision procedure were excluded because this designation may reflect biopsy rather than an intervention with curative intent. Patients who underwent radiation therapy alone were excluded due to a small sample size, as were those with unknown treatment regimens.

### 2.3. Variables

Patient characteristics assessed included sex, age, race, insurance status, and socioeconomic status. Facility characteristics assessed included facility type and region. Clinicopathologic factors assessed were clinical tumor (T) stage, nodal involvement, and clinical metastatic (M) staging, as well as the specific treatment modality.

Age was analyzed categorically by decade (<40, 40–49, 50–59, 60–69, 70–79, and ≥80 years). Self-reported racial and ethnic demographics were categorized according to the United States national census standards (Caucasian, African-American, Hispanic, Asian/Pacific Islander, and other/unspecified) [16]. Insurance status was also assessed categorically (private/managed care, uninsured, Medicaid, Medicare, and other government insurance). Socioeconomic status (low, medium, and high) was ascertained from zip-code data as a representative measure of income and educational attainment [16].

Facilities were assessed categorically (Comprehensive Community Cancer Program, Community Cancer Program, Academic Research Program, or Integrated Network Cancer Program) as defined by the American College of Surgeons Commission on Cancer, in consideration of services provided, institutional structure, and participation in research and residency training [17]. Facility location was determined in accordance with the nine United States regional boundaries established by the national census [18].

Tumor factors including cT stage (cT1, cT2, cT3, cT4, cT unspecified), clinical node (cN) stage (cN0, cN1, cN2, cN unspecified), and clinical metastasis (cM) stage (cM0, cM1, cM unspecified) were assessed categorically. Treatment modality was analyzed categorically (no treatment, chemotherapy, surgery, chemotherapy plus surgery, chemoradiotherapy, and chemoradiation plus surgery).

### 2.4. Statistical Analysis

Adjusted Cox proportional hazards regression analysis was performed to assess the aforementioned categorical variables as individual predictors of mortality in PSCCK. A Kaplan–Meier analysis was performed to determine overall median survival in all patients and then repeated to exclude patients in the “no-treatment” category. Overall survival was defined as the time from diagnosis to last follow-up (last known date alive or date of last contact). A Log Rank (Mantel-Cox) test of equality was employed to assess survival distributions for the different modalities of treatment.

All analyses were performed using SPSS version 25.0 (IBM Corp., Armonk, NY, USA). Tests were performed two-sided with 95% confidence intervals (CI), and *p*-values < 0.05 were reported as significant.

## 3. Results

### 3.1. Sample Characteristics

A total of 110 patients met the criteria for inclusion. There was a near-equal representation of patients by sex (47:53% female:male), and most patients were older (77% ≥60 years of age) and self-identified as Caucasian (86%) (Table 1). The largest subgroups for each tumor factor were unspecified cT (54%), unspecified cN (45%), and cM0 (50%) (Table 2). The most common treatment modality was surgery alone (26%), and 24 patients (22%) received no treatment.

### 3.2. Predictors of Mortality

Significant predictors of PSCCK mortality included female sex (Hazard Ratio (HR) 2.02 (95% CI 1.02–3.99), *p* = 0.043), age 60–69 years (HR 3.50 (1.08–11.35), *p* = 0.037), treatment at an Integrated Network Cancer Program (HR 2.80 (1.02–7.71), *p* = 0.046), stage cM1 (HR 2.23 (1.03–4.83), *p* = 0.043), and no treatment (HR 3.30 (1.21–9.05), *p* = 0.020) (Table 3). Trends toward significance were also observed in patients insured by Medicaid (HR 3.76 (0.85–16.52), *p* = 0.080), located in the East North Central region (HR 2.39 (0.92–6.22), *p* = 0.075), and with stage cN1 (HR 2.46 (1.00–6.04), *p* = 0.050). Significant independent protective factors were high socioeconomic status (HR 0.33 (0.12–0.99), *p* = 0.048) and treatment at an Academic Research Program (HR 0.39 (0.19–0.83), *p* = 0.015).

### 3.3. Survival Analysis

Kaplan–Meier analysis revealed an estimated median overall survival time of 9.31 (95% CI 7.28–10.98) months with a significant difference in survival by treatment modality (*p* = 0.010). The estimated median survival was 1.64 (0.64–2.64) months in the “no treatment” group, 10.02 (7.22–12.82) months with chemotherapy alone, 9.00 (0.00–20.99) months with surgery alone, 13.50 (9.42–17.58) months with chemotherapy plus surgery, 9.36 (6.46–12.26) months with chemoradiotherapy, and could not be determined for those receiving chemoradiotherapy plus surgery (*n* = 4). Repeat analyses upon removal of the “no treatment” (*n* = 24) subgroup rendered an estimated overall median survival time of 10.28 (7.75–12.81) months (*n* = 86) with no significant difference in estimated survival across remaining treatment modalities (*p* = 0.214) (Table 4).

## 4. Discussion

In view of the rarity of PSCCK, there remains a paucity of information characterizing this malignancy, precluding risk stratification and therapy optimization. The present NCDB analysis, to our knowledge, constitutes the single largest PSCCK cohort reported to date.

The present analysis extends upon the existing corpus of knowledge surrounding the clinical profile of patients with PSCCK. In accordance with recently published data from the Surveillance, Epidemiology, and End Results registry (SEER), which notably include a comprehensive analysis of extrapulmonary SCCs as well as a descriptive analysis specific to PSCCK, PSCCK has been characterized as a disease of the elderly [19,20]. Consistently, the majority of patients in our analysis presented during or after the seventh decade of life. Furthermore, although only statistically significant among patients age 60–69 years, the present analysis suggests that advanced age confers an increased risk for mortality in PSCCK. Interestingly, while prior literature suggests a female predominance in PSCCK [13,14], this study reports a sex disparity in mortality rather than prevalence. Specifically, while this study was comprised of a near-equal number of females and males, females incurred a two-fold increased risk of mortality compared to males.

According to 2010 United States census data, Caucasians appeared to be overrepresented in the current study sample compared to the general United States population (86% vs. 77%), whereas African-American and Hispanic patients were underrepresented (7% vs. 13% and 4% vs. 18%, respectively) [16]. These findings should be interpreted in view of the fact that race-based sampling bias in the NCDB has been previously reported [21].

Notably, the present analysis also revealed significant differences in mortality risk by treatment setting and socioeconomic factors. The protective effect of an Academic Research Center on survival is consistent with prior cancer outcomes studies [22,23] and perhaps attributable to the fact that clinicians at higher-volume referral centers may garner more experience and comfort in managing rare malignancies compared to physicians at lower-volume facilities [24]. The significant and trending mortality risks associated with treatment at an Integrated Network Cancer Program and treatment in the East North Central region, respectively, merits further study. The protective effect of high socioeconomic status may be an important epiphenomenon of resource disparities in PSCCK outcomes.

The findings of the present study are also highly relevant with respect to previous literature on survival as a function of PSCCK clinical staging. In separate analyses, Majhail et al. and Lee et al. reported no differences in survival between patients with limited-stage and extensive-stage PSCCK [13,14]. However, in accordance with contemporary characterization of pulmonary SCC, the aforementioned studies defined the limited stage as a tumor confined to the kidney or regional lymph nodes [13,14,20,21]. In the present study, not only was cM1 a significant predictor of mortality, but a trend toward significance was also observed among those with stage cN1 (“limited stage”), suggesting that previous negative findings may be attributable to the combination of two unique patient subsets [13,14]. Stated alternatively, it may be that any extension outside of the kidney decreases survival, such that it may be meaningful to instead classify regional lymph node involvement and kidney-limited disease as distinct categories. Although cN0 disease was present in a minority of patients, the apparent survival advantage of cN0 disease is of particular interest in view of the trend toward more active surveillance and renal biopsies, as this clinical stage will presumptively constitute a greater proportion of PSCCK cases in the future. Further research is needed in this regard, particularly in view of the absence of significance in cN2 as an independent predictor of mortality.

Consistent with outcomes data from extrapulmonary primary SCC [25,26,27], cisplatin-based chemotherapy regimens were previously shown to carry a significant survival benefit in PSCCK [13,14]. In this study, initial survival analysis suggested a difference in survival across different treatment modalities; however, this finding was likely attributed to poorly estimated median survival times among patients in the no treatment subgroup, and significance failed to persist upon exclusion of this subset of patients. Importantly, the nature of the present study design precluded further characterization of the “no treatment” subgroup. Analysis of this subgroup was obfuscated by the group’s sub-two-month estimated median survival time, which may have been a true product of the absence of cancer treatment or rather a function of advanced-stage disease at initial diagnosis and immediate palliative care.

Irrespective of specific treatment modality, no median survival estimate exceeded 14 months from the time of initial diagnosis, underscoring the aggressive nature of PSCCK and the need for immediate and aggressive intervention. However, even among those patients who did receive treatment, significant heterogeneity was observed with respect to specific treatment regimens. It stands to reason that such differences in management reflect the fact that a standard of care for PSCCK has not been established in relevant clinical guidelines for kidney cancer [28]. Although no significant differences in survival were observed across individual treatment modalities, multimodal therapies likely merit particular investigative attention in view of growing evidence supporting their use in treating other primary small cell malignancies of the genitourinary tract [29].

The present study is subject to the inherent limitations of an NCDB analysis, including potential racial, socioeconomic, and geographic sampling bias [20,30]. Consistent with other registry studies, the study design is retrospective and observational, and the present results assume accurate and consistent variable coding. The aforementioned heterogeneity in treatment regimens, as well as the relatively high clinical stage and decentralized pathologic review, reflect additional limitations of the NCDB which directly affect the interpretation of the present study results. Additionally, the NCBD does not report specific chemotherapy regimens or treatment frequency, nor does it detail follow-up frequency. Data pertaining to specific comorbidities and functional status could not be taken into account. The small number of patients receiving multimodal treatment precluded further stratification based on whether radiotherapy and/or chemotherapy were administered before or after surgery. Notwithstanding these limitations, these findings constitute the largest cohort of PSCCK patients to date, and stand to help consolidate current knowledge on this exceedingly rare disease.

## 5. Conclusions

PSCCK is a rare and aggressive malignancy with a median survival time of less than one year. No significant difference in estimated median survival was observed across individual treatment modalities. The present study findings should be interpreted in view of several other recent related publications, including those derived from the SEER database and those describing single-institution experiences with PSCCK, which cumulatively serve to further characterize the current treatment landscape of PSCCK in the United States. Future studies that correlate clinical tumor staging with specific treatment modalities are needed to optimize and individualize management.

## Figures and Tables

**Table 1:** Patient and facility characteristics.

Characteristic	Frequency (%)
**Sex**	
Male	58 (52.7)
Female	52 (47.3)
**Age (years)**	
18–39	4 (3.6)
40–49	8 (7.3)
50–59	13 (11.8)
60–69	27 (24.5)
70–79	31 (28.2)
≥80	27 (24.5)
**Race**	
Caucasian	95 (86.4)
African-American	8 (7.3)
Hispanic	4 (3.6)
Asian/Pacific Islander	2 (1.8)
Other/Unspecified	1 (0.9)
**Insurance Status**	
Private/Managed Care	32 (29.1)
Not Insured	1 (0.9)
Medicaid	6 (5.5)
Medicare	64 (58.2)
Other Government Insurance	3 (2.7)
Unknown	4 (3.6)
**Socioeconomic Status**	
Low	37 (33.6)
Middle	32 (29.1)
High	37 (33.6)
Unknown	4 (3.6)
**Facility Type**	
Comprehensive Community Cancer Program	48 (43.6)
Community Cancer Program	11 (10.0)
Academic Research Program	37 (33.6)
Integrated Network Cancer Program	10 (9.1)
Censored *	4 (3.6)
**Facility Region**	
South Atlantic	29 (26.4)
New England	5 (4.5)
Middle Atlantic	9 (8.2)
East North Central	21 (19.1)
East South Central	5 (4.5)
West North Central	9 (8.2)
West South Central	8 (7.3)
Mountain	7 (6.4)
Pacific	13 (11.8)
Censored *	4 (3.6)

Note: values reported as frequency (%). * Per NCDB coding guidelines, patients <40 years of age have data censored for facility type and facility region to maintain privacy.

**Table 2:** Clinicopathologic and treatment characteristics.

Characteristic	Frequency (%)
**Clinical T Stage**	
cT1	14 (12.7)
cT2	8 (7.3)
cT3	16 (14.5)
cT4	13 (11.8)
cT unspecified	59 (53.6)
**Clinical N Stage**	
cN0	27 (24.5)
cN1	30 (27.3)
cN2	4 (3.6)
cN unspecified	49 (44.5)
**Clinical M Stage**	
cM0	55 (50.0)
cM1	51 (46.4)
cM unspecified	4 (3.6)
**Treatment**	
No Treatment	24 (21.8)
Chemotherapy	19 (17.3)
Surgery	29 (26.4)
Chemotherapy + Surgery	22 (20.0)
Chemoradiotherapy	12 (10.9)
Chemoradiation + Surgery	4 (3.6)

Note: values reported as frequency (percent).

**Table 3:** Cox proportional hazards regression to identify independent predictors of increased risk of mortality.

Predictor	Hazard Ratio (95% CI)	*p*-Value
**Sex**		
Male	1 (Reference)	----
Female	2.02 (1.02–3.99)	0.043 *
**Age (years)**		
50–59	1 (Reference)	----
18–39	0.90 (0.14–5.69)	0.912
40–49	0.58 (0.10–3.46)	0.550
60–69	3.50 (1.08–11.35)	0.037 *
70–79	1.07 (0.27–4.16)	0.923
≥80	2.62 (0.68–10.04)	0.161
**Race**		
Caucasian	1 (Reference)	----
African-American	1.00 (0.27–3.70)	0.996
Hispanic	2.75 (0.63–12.03)	0.180
Asian/Pacific Islander	0.44 (0.02–8.29)	0.581
Other/Unspecified	5.66 (0.14–234.76)	0.362
**Insurance Status**		
Private/Managed Care	1 (Reference)	----
Medicaid	3.76 (0.85–16.52)	0.080
Medicare	1.14 (0.46–2.82)	0.782
Other Government Insurance	2.70 (0.25–28.85)	0.411
Unknown	0.78 (0.12–5.02)	0.790
**Socioeconomic Status**		
Low	1 (Reference)	----
Middle	0.50 (0.21–1.16)	0.106
High	0.33 (0.12–0.99)	0.048 *
Unknown	1.88 (0.33–10.68)	0.477
Facility Type **		
Comprehensive Community Cancer Program	1 (Reference)	----
Community Cancer Program	0.44 (0.12–1.56)	0.202
Academic Research Program	0.39 (0.19–0.83)	0.015 *
Integrated Network Cancer Program	2.80 (1.02–7.71)	0.046 *
Facility Region **		
South Atlantic	1 (Reference)	----
New England	1.03 (0.22–4.73)	0.970
Middle Atlantic	3.03 (0.65–14.17)	0.159
East North Central	2.39 (0.92–6.22)	0.075
East South Central	0.69 (0.12–3.98)	0.682
West North Central	1.70 (0.34–8.45)	0.520
West South Central	0.34 (.09–1.25)	0.104
Mountain	1.28 (0.33–4.92)	0.720
Pacific	1.76 (0.55–5.67)	0.343
**Clinical T Stage**		
cT1	1 (Reference)	----
cT2	0.77 (0.13–4.46)	0.770
cT3	0.71 (0.21–2.37)	0.573
cT4	1.25 (0.35–4.49)	0.737
cT unspecified	0.73 (0.24–2.24)	0.577
**Clinical N Stage**		
cN0	1 (Reference)	----
cN1	2.46 (1.00–6.04)	0.050
cN2	0.60 (0.08–4.48)	0.618
cN unspecified	1.42 (0.53–3.81)	0.490
**Clinical M Stage**		
cM0	1 (Reference)	----
cM1	2.23 (1.03–4.83)	0.043 *
cM unspecified	2.33 (0.39–13.99)	0.357
**Treatment**		
Chemotherapy	1 (Reference)	----
No Treatment	3.30 (1.21–9.05)	0.020 *
Surgery	1.79 (0.49–6.48)	0.378
Chemotherapy + Surgery	0.90 (0.35–2.31)	0.818
Chemoradiotherapy	2.07 (0.76–5.64)	0.153
Chemoradiation + Surgery	0.29 (0.04–2.16)	0.228

Note: * denotes statistical significance. ** Per NCDB coding guidelines, patients <40 years of age are censored for facility type and region to maintain privacy, and thus were dropped from the model for these variables only.

**Table 4:** Median months survival by treatment regimen.

Treatment	Estimate	Std. Error	95% Confidence Interval
Chemotherapy alone	10.020	1.429	7.219–12.821
Surgery alone	9.000	6.118	0.000–20.992
Surgery + Chemotherapy	13.500	2.081	9.421–17.579
Chemoradiotherapy	9.360	1.481	6.457–12.263
Surgery + Chemoradiotherapy	12.650	---	---
Overall	10.280	1.289	7.754–12.806

## Data Availability

All data for the present analysis have been made publicly available.
